# Prevalence of *Campylobacter* spp. in broilers in North Lebanon

**DOI:** 10.14202/vetworld.2023.322-328

**Published:** 2023-02-17

**Authors:** Rana Awada, Ghassan Ghssein, Ali El Roz, Mona Farhat, Nada Nehme, Hussein F. Hassan

**Affiliations:** 1 Department of Biology, Faculty of Sciences, Lebanese University, Beirut, Lebanon; 2 Department of Laboratory Sciences, Faculty of Public Health, Islamic University of Lebanon, Beirut, Lebanon; 3 Department of Biological and Chemical Sciences, School of Arts and Sciences, Lebanese International University, Beirut, Lebanon; 4 Department of Environmental Sciences and Natural Resources, Faculty of Agricultural Engineering and Veterinary Medicine, Lebanese University, Dekwaneh, Mount Lebanon, Lebanon; 5 Department of Natural Sciences, School of Arts and Sciences, Lebanese American University, Beirut, Lebanon

**Keywords:** broilers, *Campylobacter*, campylobacteriosis, faeco-prevalence, Lebanon

## Abstract

**Background and Aim:**

Great attention has been given recently to the prevalence of different *Campylobacter* spp. in poultry since the latter are considered the major contributing reservoir of human campylobacteriosis. In Lebanon, the occurrence of campylobacteriosis in humans is high. The aim of our first-of-its-kind study in the country was to estimate the prevalence of *Campylobacter* spp. in broilers from a convenient sample of farms in North Lebanon.

**Materials and Methods:**

One hundred twenty-five fecal samples were collected from 25 broiler farms, which were selected, examined, and classified according to their biosecurity level and rearing system. All samples were subjected to qualitative microbiological culture testing and polymerase chain reaction (PCR) assays to detect *Campylobacter* spp.

**Results:**

Despite the reported use of antibiotics, cell culture and PCR were positive for 44% and 88%, respectively. This implies that this bacterium is resistant to antibiotics used on the farms. Furthermore, *Campylobacter* infection rate was higher in open (92%) than in closed (85%) system farms. All farms with poor biosecurity measures, and 82% of farms with good biosecurity measures had *Campylobacter* infections, and the difference was significant (p < 0.05).

**Conclusion:**

Our results show that campylobacteriosis was found prevalent among broilers in North Lebanon, making them potential carriers of *Campylobacter* spp. Future studies should include antibiotic susceptibility testing to check the susceptibility pattern of isolates.

## Introduction

*Campylobacter* spp. are considered globally as a major cause of bacterial gastroenteritis, with an increased recurrence in Lebanon, especially over the last few years [1–4]. *Campylobacter* spp. are zoonotic bacteria having birds and canines as reservoirs [5]. *Campylobacter* is usually transmitted to humans after the ingestion of contaminated water or feed [6]. Once ingested, *Campylobacter* causes an inflammation of the colon accompanied by an infiltration of mucosa with neutrophils and lymphocytes [7] and a toxin-releasing inflammatory response in the host, causing campylobacteriosis [8]. Clinical signs of campylobacteriosis range from mild, self-limiting enteritis to bloody mucoid diarrhea that can be accompanied by intermittent vomiting, pyrexia, and anorexia in acute campylobacteriosis [9–11]. In rare cases, this infection can lead to a neurological disorder called Guillan-Barré Syndrome [12]. *Campylobacter* spp. are mesophilic germs [13] and considered as auxotrophic bacteria because of their limited ability to utilize available nutrients and of their slow growth rate (several days of culture for colonies to appear) in comparison with other intestinal bacteria [14]. In case of campylobacteriosis suspicion, fresh feces should be collected in sterile containers and stored in refrigerated conditions [7]. Then, identification can be made through direct microscopic examination of fecal culture on *Campylobacter* specific media or by polymerase chain reaction (PCR) based methods [15, 16]. The fine bacilli *Campylobacter* are recognized by sequencing of 16S rRNA. Out of all *Campylobacter* spp., *Campylobacter jejuni*, *Campylobacter coli* and *Campylobacter upsaliensis* are of major interest to public health [9].

Despite its extreme vulnerability, *Campylobacter* is able to survive in the broiler farm and can be passed from one rearing cycle to the next [17]. Chickens are coprophagic; thus, poor biosecurity and an intensive production system are the main factors in the spread of infection from infected chicken to others [18]. In fact, 1 week is enough for intestinal colonization of all the chickens within the same farm. This infection will last until the age of slaughter [19]. Latency period lasts from the time of hatching until around 2 weeks of age, during which *Campylobacter* cannot be detected even if the organism is infected [20]. Fluoroquinolones, macrolides (erythromycin), aminoglycosides (gentamycin), and tetracyclines are frequently used to treat campylobacteriosis in animals and humans [2]. People working in chicken slaughterhouses are at high risk of having *Campylobacter* infection, but this infection is often asymptomatic [21]. The risk of human campylobacteriosis arising from food sources has been extensively studied during the past years [2, 22]. In contrast, the risk of environmental exposure to fecal material from livestock and pets is less studied [23, 24]. The previous study on wild birds and pet animals have identified a relation between *Campylobacter* populations from birds and human campylobacteriosis that cause enormous fecal contamination of the environment [25].

In Lebanon, food safety has been at stake for the last two decades [26–28]. Different investigations were done regarding human campylobacteriosis, in particular, prevalence and clinical manifestation among the Lebanese population [2], and prevalence of this pathogen in different types of foods of animal origin [2, 29–31was extracted using 1]. In households and farms in Lebanon, *Campylobacter* remains an underdiagnosed pathogen.

Therefore, this study aimed to estimate the prevalence of *Campylobacter* spp. in farm broilers in Lebanon.

## Materials and Methods

### Ethical approval

The study was approved by the Institutional Review Board at the Lebanese American University. IRB approval number: LAU.SAS.HH1.4/2019. A licensed veterinarian was present with a comprehensive questionnaire including frequency of antibiotic use, that had been completed for each farm.

### Study period and location

The study was conducted from April to September 2019. Samples were collected from North Lebanon and analyzed in the Microbiology Laboratory at the Lebanese Agricultural Research Institute.

### Sample collection

The study was conducted in a convenient sample of 25 broiler farms located in two districts of North Lebanon: 13 farms were in a closed system with a high level of biosecurity, and 12 farms were in an open system. The biosecurity level was assessed using a questionnaire adapted from the literature [29, 32, 33]. In total, these farms produce 2.5 million broilers per year, with capacities varying from 10,000 to 30,000 broilers per rearing cycle.

One hundred twenty-five caeca were collected from 25 broiler farms (5 caeca from each farm were taken randomly). Five millimeters of buffer were added to the Sterile Transwabs to collect samples. Then, they were refrigerated and directly transported to the microbiology laboratory at the Lebanese Agricultural Research Institute, the accredited laboratory in Lebanon, within a maximum of 4 h.

### Media preparation and bacterial culture

Charcoal cefoperazone deoxycholate modified agar base media (mCCDA) was prepared according to the manufacturer’s instructions (HiMedia, Mumbai, India). Briefly, 22.4 g of powder was suspended in sterile distilled water and brought to boiling temperature to dissolve. The mixture was then sterilized in the autoclave and kept to cool down to 50°C. One vial of the *Campylobacter* CCDA selective supplement containing two antibiotics, cefoperazone and amphoterricin B, was added and carefully mixed in sterile Petri dishes. Using the direct plating technique, each sample was swabbed on a dried Petri dish containing the mCCDA media using the 4 quadrants method. The dishes were then incubated at 37°C for 48 h in microaerophilic conditions using atmospheric generators (GENbox Microaer, bioMérieux, France).

### Identification, isolation, and purification

Suspected typical light gray colonies were examined under a microscope after Gram-negative staining. Colonies containing “S” shaped or spiral bacteria with darting motility were reported as *Campylobacter* spp. and thus isolated and re-cultured in a new Petri dish containing mCCDA for purification. New dishes were incubated for 48 h in the same conditions (microaerophilic atmosphere at 37°C). The new colonies were re-examined under the microscope for further confirmation of the presence of *Campylobacter* spp. as mentioned above. Once confirmed, bacteria were moved to a third Petri dish containing the same media (mCCDA) with added peptone water for bacterial growth enhancement after bacterial plating with a spreader. Then, the Petri dish was incubated at 37°C for 48 h [34, 35].

### Bacterial DNA extraction

For bacterial DNA extraction, Quick-DNA™ Fecal/Soil Microbe Miniprep Kit by ZYMO Research was used (Zymo Research, Irvine, CA, USA). DNA was extracted using 150 μL of each collected sample following the manufacturer’s instructions. Samples were directly added to a ZR BashingBead™ Lysis Tube and efficiently lysed using a bead beater homogenizer. This technique does not require the use of any organic denaturants or proteinases that could harm the DNA. Zymo-Spin™ Technology was then applied to isolate DNA, which was subsequently filtered to remove any substance that inhibits PCR procedure, such as humic acids and polyphenols. Purified DNA was stored at –20°C and used for PCR in less than a week after DNA quantification using a nanodrop (BioSpec-nano Micro-Volume Spectrophotometer by Shimadzu).

### Campylobacter detection using PCR method

Polymerase chain reaction method is used in molecular biology to detect *Campylobacter* spp. by allowing the amplification of a targeted DNA sequence using specific primers. Primers used in this study were the *16S rRNA* gene, specific for the *Campylobacte*r spp. with the following sequence:

Forward primer: 5’-GGAGGCAGCAGTAGG GAATA- 3’

Reverse primer: 5’-TGACGGGCGGTGAGTAC AAG- 3’

Using MasterMix (Biomérieux), amplification reactions were performed in a mixture containing water, Taq buffer, deoxynucleotide triphosphate, Taq Polymerase (Taq PCR core kit, Qiagen) and the above-mentioned primers. Amplification reactions were carried out using a DNA thermal cycler (Veriti 96 Well Thermal Cycler, Applied Biosystems, USA). The amplification generated 1044 bp DNA fragments corresponding to the *Campylobacter* genus. Amplified products were identified by electrophoresis in a 1% (weight/volume) agarose gel in TBE buffer 1× (Gibco^®^ by life technologies™, Thermo Fisher Scientific, Vilnius, Lithuania) along with a 1Kb Plus ladder (GeneRuler 100bp DNA Ladder, Thermo Fisher Scientific), subsequently stained with ethidium bromide and exposed to UV light using UVP ( GelDoc-it™ Imaging System, Thermo Fisher Scientific, Waltham, MA, USA).

### Statistical analysis

Statistical analysis was conducted using Statistical Package for the Social Sciences software (SPSS Inc., version 24.0, IBM Corp., NY, USA). This software was used as well for data management and cleaning. Descriptive statistics were carried out and reported as frequencies and percentages for categorical variables. The Chi *-*square test was used to assess any significant difference between the categorical variables. The significance level was set at p < 0.05 for all statistical analyses.

## Results

### Fecal culture results

Following the direct fecal culture on mCCDA media for 48 h, typical *Campylobacter* colonies, distinguished by their small gray point shape, were examined under an optical microscope to study their shape and motility. The pink color because of the Gram stain technique was considered as a positive *Campylobacter* sample. Positive colonies were then isolated and cultured by adding peptone water for maximum proliferation on an mCCDA new Petri dish. After incubation, pure cultures of *Campylobacter* were obtained. Out of the 25 assessed farms, 11 (44%) showed the presence of *Campylobacter* spp. in samples.

### Bacterial identification by PCR

*Campylobacter* is a bacterium that could be present in samples without being able to be detected by culture. Under unfavorable conditions of growth, these microorganisms have the ability to form viable but non-culturable cells. The death of bacteria, due to the use of antibiotics prevents their detection under a microscope. In this regard, and to avoid false negative cases by bacterial culture, PCR was performed to detect bacterial DNA, which would indicate the presence of bacteria in broilers.

Results obtained from PCR showed that the total number of *Campylobacter*-positive farms increased, compared to the routine culture identification technique, from 11 (44%) to 22 (88%). The molecular weight marker was used to assess all parameters. Sample with a molecular weight equal to the expected PCR product size 1044 bp was judged or considered as positive [36].

### Relationship between the frequency of antibiotics use on broiler farms and *Campylobacter spp*. resistance

**Figure-1:**
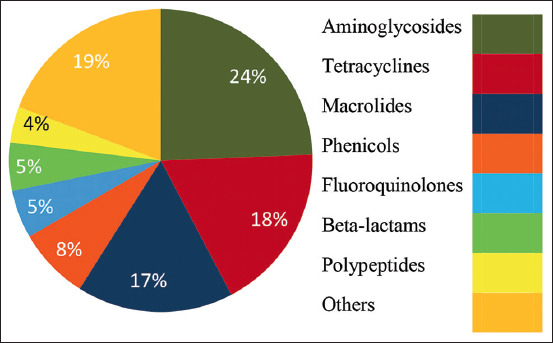
Percentage of different classes of antibiotics used in the 25 farms.

**Figure-2:**
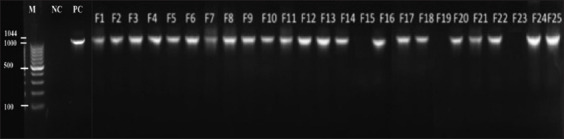
Results of the polymerase chain reaction on agarose Gel. M=Marker, PC=Positive control, NC=Negative control, S=Sample, F=Farm.

*Campylobacter* becomes resistant to antibiotics if they are frequently used for prophylaxis. For this reason, the relationship between frequency of antibiotics used on broilers farm and *Campylobacter* spp. resistance was assessed. A total of ten different antibiotics belonging to 7 classes were reported to be used. Fosfomycin was the most widely used antibiotic in 60% of the farms, followed by Neomycin (52%) (Figure-1). The number of antibiotics administered on a single farm during a single production cycle varied from 1 to 5, with an average of 3 antibiotics per farm. These antibiotics mainly belong to aminoglycosides (24%), tetracyclines (18%), and macrolides (17%) classes, while fosfomycin, which inhibits the synthesis of the bacterial cell wall, does not belong to any class (Figure-2). Usually, the use of antibiotics decreases the proportion of infected farms [37]. Despite the use of antibiotics in the visited farms, *Campylobacter* contamination remained high (88%), suggesting that this bacterium is already resistant to the antibiotics used in these farms.

### Relationship between applied biosecurity measures, farming system, and farm infection

Biosecurity is the first line of defense against any disease on a farm. It is linked to good disinfection between production cycles of broilers, and to a good structure of the farm and protection against contact with wild animals potentially carrying *Campylobacter*, especially birds and insects. One of the most important biosecurity factors is the displacement of farmers who must be responsible in terms of implementing measures, such as wearing clean clothing and boots, and restricting their movement to other livestock. Table-1 shows a significant association between the farming system used (open or closed) and the level of biosecurity applied (good or bad) (p < 0.05). About 92% of closed system farms were found to enforce biosecurity rules, which is not always the case in open system farms (42%) (results are not shown).

**Table-1:** Association between the farming system used (open or closed) and the level of biosecurity applied (good or bad).

System	Biosecurity		Total	p-value

	Bad	Good		
Closed				
n	1	12	13	0.007
%	8	92	100	
Open				
n	7	5	12	0.0025
%	58	42	100	

Table-2 shows that the *Campylobacter* infection rate was higher in open (92%) than in closed system (85%) farms, but the difference was not significant (p > 0.05). Table-3 shows that all farms with poor biosecurity (8/8), and 75% of farms with good biosecurity (14/17) had *Campylobacter* infections, and the difference was significant (p < 0.05).

**Table-2:** Faeco-prevalence of *Campylobacter* spp. in open and closed farms.

System	*Campylobacter*		Total	p-value

	Negative	Positive		
Closed				
n	2	11	13	0.588
%	15	85	100	
Open				
n	1	11	12	0.99
%	8	92	100	

**Table-3:** Faeco-prevalence of *Campylobacter* spp. in farms with good and bad biosecurity.

Biosecurity level	*Campylobacter*		Total	p-value

	Negative	Positive		
Good				
n	3	14	17	0.005
%	18	82	100	
Bad				
n	0	8	8	0.0065
%	0	100	100	

## Discussion

Campylobacter is considered a main cause of bacterial gastroenteritis worldwide. Campylobacteriosis is a zoonosis transmitted to humans by animals or derived products [38]. Researchers from “Emerging Pathogens Institute” of the University of Florida in United States estimated that 31 foodborne pathogens are responsible for 9.4 million cases of human infections each year in United States, leading to 55,961 hospitalizations and 1,351 deaths. Among all these cases, 39% are associated with bacteria, mainly *Campylobacter* spp., *Salmonella* spp., and *Clostridium perfringens*. The most abundant zoonotic bacteria in European Union is campylobacter. Indeed, 190,566 cases of Campylobacter infections were reported in 2008 [38].

To illustrate the importance of campylobacter infections in Lebanon, it was interesting to detect the presence of this bacteria in chicken broilers, the main reservoir of such pathogen. Our study reported that the faeco-prevalence of *Campylobacter* spp. was 88% in broilers, with significantly high prevalence in farms with poor biosecurity levels. These results are close to those recorded in France and in United Kingdom in 2008 (76.1% and 75.3%, respectively), but higher than what [29, 30] reported previously in Lebanon (41% and 67%, respectively). In Lebanon, as in other countries, the dispatch of broilers from the same farm can last up to 2 weeks, during which rules of biosecurity are no longer respected. This would increase the risk of infection in broilers. Detection of *Campylobacter* requires at least 48 h by direct culture with better sensitivity and reliability than its detection by PCR [39, 40, 41]. In our study, PCR was more sensitive than direct culture (Figure-2. In addition, the detection threshold for *Campylobacter* is estimated at 10^2^ colony-forming units (CFU)/g by direct culture on mCCDA and 105 CFU/g by PCR after extraction of DNA from feces [42]. By comparing both Campylobacter detection methods, our findings reported that culture alone tends to underestimate the prevalence of this bacterium, being able to detect about 50% of positive farms. This is in agreement with other studies that showed 30% of false negatives by culture compared to PCR [43]. Polymerase chain reaction results showed a rate of 88%, confirming previously published data on the search for Campylobacter of avian origin in Brazil (100% prevalence) [44], in Costa Rica (80%) [45], and in Sri Lanka (63.8%) [46].

Excessive use of antibiotics could cause culture failure in a high number of false negative cases. The high rate of positive cases suggests that *Campylobacter* is resistant to most of the antibiotics used in these farms. Our study is in line with the results obtained by Pérez *et al*. [47] in 2014, showing that the use of fosfomycin in the production of chickens has recently increased due to the emergence of bacterial resistance to other antibiotics after decades of heavy use. In our study, a high proportion of *Campylobacter* resisted the effects of different families of antibiotics. This is in line with the previous studies where a high percentage of *Campylobacter* spp. isolated from poultry are resistant to many classes of antibiotics, in particular ciprofloxacin, which is the antibiotic of choice for gastroenteritis treatment in adults, in addition to erythromycin [48]. Recent studies in Lebanon showed that *Campylobacter* collected from cecum of broilers was resistant to tetracycline and amoxicillin (95% and 40%, respectively), in addition to ciprofloxacin and erythromycin (16% and 4%, respectively) [2]. The aforementioned findings are in agreement with our results, which show that amoxicillin, ciprofloxacin, and erythromycin are the least used as opposed to tetracyclines, which are frequently used with resistance reaching 95%. In conclusion, erythromycin should be banned in breeding to preserve its efficacy in cases of human campylobacteriosis, since *Campylobacter* is already known to be resistant to ciprofloxacin worldwide [49].

Biosecurity is the first line of defense against any viral or bacterial infection. A previous qualitative study was published in UK regarding the attitudes and perceptions of broiler farm workers, confirming the importance of biosecurity in the control of *Campylobacter* [50]. Lack of biosecurity surveillance programs is the main cause of the spread of *Campylobacter*. The application of strict disinfection and biosecurity programs in developed countries has made it possible to delay the age of infection in broilers up to more than 20 days in comparison with developing countries, such as in Jordan, where the age of infection is 8 days on average [33]. Biosecurity rules that are directly involved in the prevention of *Campylobacter* infection include restricting entry and exit movements of workers, changing clothes and boots at the entrance to the farm, and preventing any contact between the interior of the farm and its environment, which is rich in *Campylobacter*, such as birds, rodents, and insects, especially mealworms [51]. Good management of the farm as well as education of farmers and workers, has shown to be necessary to reduce the risk of *Campylobacter* spread [52]. Indeed, a British study found that one-third of contamination could be avoided if good biosecurity measures were applied and if partial depopulation was avoided [53]. In our study, the presence of mealworms was noted during the visits to farms, which seem to escape insect control measures, despite good biosecurity practices. These insects are potential reservoirs of *Campylobacter* [54, 55], explaining in part the high proportion (75%) of positive farms where biosecurity measures are applied. The application of good biosecurity measures appears to be a determining factor in preventing the spread of *Campylobacter*, yet two factors still need to be reconsidered. First, insects like mealworms, are capable of harboring *Campylobacter* and can escape this high level of biosecurity. Second, the collection of broilers takes place over a period of up to 2 weeks on the same farm, and biosecurity measures are no longer applied during this period.

## Conclusion

Due to pathogenesis and high prevalence of *Campylobacter* spp., they are considered to be the leading cause of bacterial acute gastroenteritis in humans and animals around the world. Many factors contribute to the occurrence of this infection, including hygienic measures, quality of food and water provided, as well as the type of environment, whether it is indoors or outdoors. Despite the limitation of the study, which is the fact that bacterial identification based on colony morphology, motility, and Gram staining is not enough to assign isolates as *Campylobacter*, and therefore further biochemical and molecular tests must be achieved before. Our study is the first to report, by molecular tools, an overall prevalence of *Campylobacter* spp. in farm broilers in North Lebanon. Future studies must include antibiotic susceptibility testing to check the susceptibility pattern of isolates. Our study showed that the prevalence of *Campylobacter* spp. in broiler farms in Lebanon was 88%. The misuse of broad-spectrum antibiotics in farms leads to an increase in antibiotic resistance in *Campylobacter* spp. Control measures should be taken to reduce or even prevent campylobacteriosis in broiler farms and, thus, prevent its transmission to humans. In this context, owners and veterinary practitioners must play a crucial role in reducing infection occurrence and transmission of campylobacteriosis. Practices include respecting hygienic measures, improving food and water quality, and adding campylobacteriosis testing to routine work up at the veterinary clinics, especially when the patient is presented for gastro-intestinal disorders for early diagnosis and treatment. Furthermore, extensive research is necessary in Lebanon to better understand the epidemiology of *Campylobacter* spp. and its antimicrobial resistance, and thus, improving the overall health status of both animals and humans.

## Authors’ Contributions

HFH and RA: Conceptualized and designed the study, Statistical analysis, and drafted the manuscript. GG, AE, MF, and NN: Facilitated the sample collection, analyzed the samples, results, and drafted the manuscript. All authors have read, reviewed, and approved the final manuscript.
